# Effect of thermal processing on T cell reactivity of shellfish allergens - Discordance with IgE reactivity

**DOI:** 10.1371/journal.pone.0173549

**Published:** 2017-03-08

**Authors:** Jodie B. Abramovitch, Andreas L. Lopata, Robyn E. O’Hehir, Jennifer M. Rolland

**Affiliations:** 1 Department of Immunology and Pathology, Monash University, Melbourne, Victoria, Australia; 2 Department of Allergy, Immunology and Respiratory Medicine, The Alfred Hospital and Monash University, Melbourne, Victoria, Australia; 3 Department of Molecular and Cell Biology, James Cook University, Townsville, Queensland, Australia; Monash University, Australia, AUSTRALIA

## Abstract

Crustacean allergy is a major cause of food-induced anaphylaxis. We showed previously that heating increases IgE reactivity of crustacean allergens. Here we investigate the effects of thermal processing of crustacean extracts on cellular immune reactivity. Raw and cooked black tiger prawn, banana prawn, mud crab and blue swimmer crab extracts were prepared and IgE reactivity assessed by ELISA. Mass spectrometry revealed a mix of several allergens in the raw mud crab extract but predominant heat-stable tropomyosin in the cooked extract. PBMC from crustacean-allergic and non-atopic control subjects were cultured with the crab and prawn extracts and proliferation of lymphocyte subsets was analysed by CFSE labelling and flow cytometry. Effector responses were assessed by intracellular IL-4 and IFN-γ, and regulatory T (CD4^+^CD25^+^CD127^lo^Foxp3^+^) cell proportions in cultures were also compared by flow cytometry. For each crustacean species, the cooked extract had greater IgE reactivity than the raw (mud crab p<0.05, other species p<0.01). In contrast, there was a trend for lower PBMC proliferative responses to cooked compared with raw extracts. In crustacean-stimulated PBMC cultures, dividing CD4^+^ and CD56^+^ lymphocytes showed higher IL-4^+^/IFN-γ^+^ ratios for crustacean-allergic subjects than for non-atopics (p<0.01), but there was no significant difference between raw and cooked extracts. The percentage IL-4^+^ of dividing CD4^+^ cells correlated with total and allergen-specific IgE levels (prawns p<0.01, crabs p<0.05). Regulatory T cell proportions were lower in cultures stimulated with cooked compared with raw extracts (mud crab p<0.001, banana prawn p<0.05). In conclusion, cooking did not substantially alter overall T cell proliferative or cytokine reactivity of crustacean extracts, but decreased induction of Tregs. In contrast, IgE reactivity of cooked extracts was increased markedly. These novel findings have important implications for improved diagnostics, managing crustacean allergy and development of future therapeutics. Assessment of individual allergen T cell reactivity is required.

## Introduction

Shellfish, comprising crustacean and mollusc species, are a major cause of IgE-mediated adverse food reactions including anaphylaxis [1, 2]. Unlike many other food allergies, shellfish allergy predominantly affects adults and is usually lifelong [3]. There is currently no specific therapy for shellfish allergy, with patients relying on complete food avoidance to prevent reactions and adrenaline for emergency treatment of anaphylaxis. Several shellfish allergens have been identified on the basis of patient serum IgE reactivity [2, 4, 5], but studies of cellular immune reactivity of shellfish allergens are limited. The major shrimp allergen, tropomyosin, was shown to induce CD4^+^ T cell proliferation in allergic subjects and several T cell epitopes of shrimp tropomyosin and arginine kinase have been identified [6–8]. Rational design of a specific treatment requires elucidation of factors that influence development of the Th2-polarized response to shellfish allergens. Allergens are taken up by antigen presenting cells (APC) at mucosal surfaces, processed and presented as peptides complexed with MHC class II molecules to CD4^+^ T helper cells. In allergic individuals, allergen-stimulated T cells secrete IL-4, IL-5 and IL-13, Th2-type cytokines, which initiate and propagate the allergic IgE-mediated immune response [9, 10]. On subsequent exposure to food allergens, mast cells and basophils are activated by allergen cross-linking of surface-bound specific IgE, releasing a cascade of inflammatory mediators that elicit the clinical manifestations of food allergy. Adding complexity, other cell types including type 2 innate lymphoid cells (ILC2s) and NKT cells may also play a role in shaping the immune response to allergens via their cytokine profiles [11]. Regulatory T cells (Tregs), characterized by expression of the transcription factor Foxp3, are important regulators of immune responses via direct cell-to-cell contact mechanisms or release of the regulatory cytokines IL-10 and TGF-β [12, 13]. A role for Tregs in controlling allergic immune responses, including food allergy, is suggested by reports of decreased proportions of peripheral blood Foxp3^+^ cells and impaired Treg function in food-allergic individuals [14, 15].

Food processing can influence recognition of food allergens by immune cells and the ensuing immune response [16]. Cooking can alter allergen structure via protein denaturation, aggregation and chemical modifications (e.g. Maillard reaction) [17]. These structural changes may result in allergen engagement with different receptors on immune cells (especially APC) and activation of different signalling pathways, potentially modifying allergen uptake and presentation by APC and altering the subsequent immune response [18–20]. We reported previously that cooking caused a marked increase in IgE reactivity of crustacean allergens [4, 21]. Here we report, for the first time, the characterization of crustacean-allergic and non-atopic subject peripheral blood mononuclear cell (PBMC) responses to raw and cooked extracts from four commonly ingested crustacean species. The proliferation and effector cytokine profile (IFN-γ, IL-4) of CD4^+^, CD8^+^ and CD56^+^ cells, and Foxp3^+^ Treg proportions were compared. This analysis of the cellular response to differently processed crustacean allergens will inform development of safe and effective specific immunotherapy as well as monitoring bioassays.

## Materials and methods

### Ethics statement

Informed written consent was obtained from all subjects, with ethics approvals from the Alfred Hospital Research Ethics Committee (Project number 192/07) and Monash University Human Research Ethics Committee (MUHREC CF08/0225).

### Subjects

Peripheral blood samples were obtained from eight crustacean-allergic subjects (mean age 34.5; 5 female, 3 male) and four non-atopic controls (mean age 47.8 years; 3 female, 1 male) (Table 1). Allergic subjects were recruited from the Alfred Hospital Allergy clinic on the basis of a convincing clinical history of allergy to crustaceans and positive shrimp- and crab-specific IgE (ImmunoCAP [Thermo Scientific, Uppsala, Sweden] >0.35 kU_A_/L). Non-atopic control subjects had a negative skin prick test to a panel of common aeroallergens (including house dust mite), negative shrimp- and crab-specific IgE (ImmunoCAP), no IgE reactivity to shellfish extracts by IgE immunoblotting, and no clinical history of crustacean allergy.

**Table 1:** Summary of clinical features of crustacean-allergic (A1-8) and non-atopic control (N1-4) subjects.

Subject ID	Age (yrs)	Sex	Total IgE (IU/mL)	Crab- specific IgE (kU_A_/L)	Shrimp- specific IgE (kU_A_/L)	Clinical presentation to crustaceans	
						*Symptoms*	*Known crustacean species*
A1	32	M	183	3.09	6.84	An, O	Crustaceans
A2	22	M	81	2.37	2.57	As, R, U, An, A	Crustaceans
A3	31	F	130	6.97	8.98	O	Crab
A4	49	F	579	5.55	3.63	R, H	Crustaceans
A5	39	F	345	3.82	4.23	R, An, O	Prawn, crab
A6	25	F	227	13.40	9.81	An, O	Prawn, crab
A7	32	M	581	3.81	6.98	As, U	Prawn, crab, lobster
A8	46	F	322	6.60	6.73	A, U, An	Prawn
N1	58	M	14	0.02	0.02	-	-
N2	62	F	7	0.02	0.02	-	-
N3	37	F	41	0.02	0.04	-	-
N4	38	F	4	0.01	0.01	-	-

F: female, M: male. As: asthma, R: rhinitis, A: anaphylaxis, U: urticaria, An: angioedema, O: oral/facial symptoms, H: hypotension.

### Preparation of crustacean extracts

Extracts were prepared as described previously [4]. Briefly, fresh blue swimmer crab (*Portunus pelagicus*), black tiger prawn (*Penaeus monodon*), banana prawn (*Fenneropenaeus merguiensis/indicus*) and mud crab (*Scylla serrata*) were purchased from local markets. For raw blue swimmer crab (RC1), raw mud crab (RC2), raw black tiger prawn (RP1) and raw banana prawn (RP2) extracts, the outer shell was removed and muscle collected. For cooked blue swimmer crab (CC1), cooked mud crab (CC2), cooked black tiger prawn (CP1) and cooked banana prawn (CP2) extracts, the outer shell was retained during the heating process (20 minutes immersed in boiling PBS) before its removal and muscle tissue extracted. Finely cut muscle was blended in PBS pH 7.2 and incubated overnight at 4°C with constant mixing. After centrifugation at 13,000 rpm at 4°C for 20 minutes the supernatant was collected, dialyzed against PBS and filter sterilized before storage at -80°C in aliquots. Extract protein concentrations were determined using the Bradford assay kit (Bio-Rad Laboratories, Hercules, CA) with bovine gamma globulin as a standard. All extracts were confirmed to be neither mitogenic nor toxic as described previously [22]. Endotoxin levels in all extracts were negligible (<2 EU/mL; QCL-1000 Endpoint Chromogenic LAL Assay; Lonza, Basel, Switzerland).

### Sodium Dodecyl Sulfate-Polyacrylamide Gel Electrophoresis (SDS-PAGE)

Proteins of crustacean extracts, 15 μg/lane, were separated by electrophoresis under reducing conditions using 4–12% Bis-Tris gels (NuPage, Carlsbad, CA). Pre-stained standards (1x See Blue Plus2, Invitrogen, Carlsbad, CA) were used as molecular weight markers. Proteins were resolved at 200 V for 35 minutes using an Xcell II mini-cell apparatus (Invitrogen) and stained with Coomassie brilliant blue.

### IgE immunoblot and mass spectrometry

Proteins (3 μg/lane) from raw and cooked mud crab extracts (RC2 and CC2, respectively) were separated by SDS-PAGE as above and assessed for IgE reactivity by immunoblotting using pooled shellfish-allergic serum as described previously [4, 23, 24]. IgE-reactive protein bands were excised from SDS-PAGE gels, de-stained, reduced, alkylated and digested with trypsin as reported previously [24]. Digested proteins were analysed by LC-MS/MS ion trap mass spectrometer coupled online with HPLC. Data were searched against the SwissProt database using the MASCOT search engine with Metazoa (animals) taxonomy selected using the following search parameters: missed cleavages, 1; peptide mass tolerance, + 0.6 Da; peptide fragment tolerance, + 0.3 Da; peptide charge, 2+, 3+ and 4+; fixed modifications, carbamidomethyl; variable modification, oxidation (Met).

#### IgE ELISA

IgE ELISA was based on a method described previously [4]. Briefly, wells of a 96-well EIA/RIA plate (Costar, St. Louis, MO) were coated with 100 μl crustacean extract (1 μg/mL) or PBS as a ‘no antigen’ control and incubated overnight at 4°C. The plate was blocked for 1 hour with 5% skim milk powder diluted in PBS-0.05% Tween (PBS-T) and washed in PBS-T. Serum (1:10) was added to wells and incubated for 3 hours at room temperature before washing in PBS-T. Rabbit anti-human IgE antibody (1:4000; Dako, Glostrup, Denmark) and goat anti-rabbit IgG-HRP (1:1000; Promega, Madison, WI) were added sequentially for one hour each, with washing in between with PBS-T. The plate was finally washed in PBS-T and then PBS before development with TMB substrate (Invitrogen). The reaction was terminated using 1 M HCl and the OD450 nm measured.

#### PBMC proliferation assays

PBMC were isolated from heparinized whole blood by Ficoll (Quantum Scientific VWR, Radnor, PA) gradient. PBMC (10^7^ cells/mL PBS) were labelled with 0.5 μM carboxyfluorescein succinimidyl ester (CFSE) (Life Technologies) and cultured at 2.5 x 10^5^ cells/well in 96-well microplates with culture medium alone (‘no antigen’ negative control), tetanus toxoid as positive control (TT; 15 lfu/mL; Statens Serum Institute, Copenhagen, Denmark) or each of the eight crustacean extracts (50 and 100 μg/mL). Culture medium comprised RPMI-1640 (Life Technologies, Carlsbad, CA) supplemented with 2 mM L-glutamine (Life Technologies), 100 U/mL penicillin-streptomycin (Life Technologies) and 5% heat-inactivated AB^+^ human serum (Sigma-Aldrich, St Louis, MO). All conditions were plated in duplicate. Cultures were incubated for 7 days at 37°C in 5% CO_2_.

#### Cell staining and flow cytometry

For intracellular cytokine staining, cultured cells were re-stimulated with 10 ng/mL phorbol 12-myristate 13-acetate (PMA; Sigma-Aldrich) and 250 ng/mL ionomycin (I; Sigma-Aldrich) for 5 hours in the presence of Brefeldin A (BFA; 10 μg/mL; Sigma-Aldrich) for the last 4 hours at 37°C in 5% CO_2_ prior to antibody staining. Cells from duplicate cultures were pooled and incubated with an antibody cocktail containing aqua live/dead dye (Life Technologies) and fluorochrome-labelled anti-human CD4, CD8 and CD56 (BD Biosciences, San Jose, CA) for 30 minutes at 4°C in the dark. Cells were then fixed with 1% paraformaldehyde and stained with fluorochrome-labelled anti-human IL-4 and anti-human IFN-γ (BD Biosciences) diluted in 0.3% saponin (Sigma, St Louis, MO) for 1 hour at 4°C in the dark. For Treg analysis, cells were stained with aqua live/dead dye and an antibody cocktail containing fluorochrome-labelled anti-human CD4, CD25 and CD127 (BD Biosciences) for 20 minutes at room temperature in the dark. Cells were then fixed and permeabilized (fixation/permeabilization buffer; eBioscience, San Diego, CA) for 30 minutes at 4°C in the dark, blocked with 2% rat serum (Sigma-Aldrich) in 1X permeabilization buffer (eBioscience) for 15 minutes at 4°C in the dark and incubated with fluorochrome-labelled anti-human Foxp3 (eBioscience) for 30 minutes at 4°C in the dark. Data were acquired using an LSR-Fortessa (BD Biosciences) and analysed using FlowJo 7.6.4 (TreeStar) software.

### Statistical analysis

Graphpad Prism 6 software (GraphPad Software; La Jolla CA, USA) was used for statistical analyses and a p-value of <0.05 was considered significant. Statistical methods are indicated in Figure legends.

## Results

### Altered protein profiles in cooked compared with raw crustacean extracts

**Fig 1:**
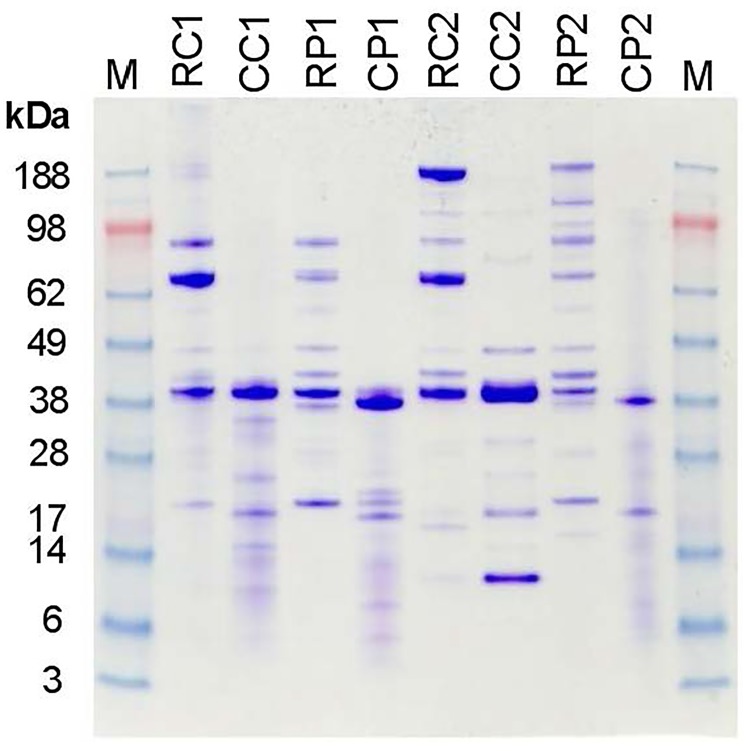
SDS-PAGE of crustacean extracts. SDS-PAGE of proteins within crustacean extracts stained with Coomassie brilliant blue is shown. M, molecular weight markers (kDa). Extracts: RC1, raw blue swimmer crab; CC1, cooked blue swimmer crab; RP1, raw black tiger prawn; CP1, cooked black tiger prawn; RC2, raw mud crab; CC2, cooked mud crab; RP2, raw banana prawn; CP2, cooked banana prawn.

SDS-PAGE analysis of raw and cooked crustacean extracts showed a range of proteins from approximately 5–188 kDa (Fig 1). In raw extracts, protein bands were observed that corresponded with the molecular weights of documented crustacean allergens—arginine kinase (40 kDa), tropomyosin (38–39 kDa), and myosin light chain, sarcoplasmic calcium binding protein and troponin C (21 kDa). The cooked extracts showed marked differences from the raw extracts—there was a loss of higher molecular weight proteins, an increase in lower molecular weight bands and protein smearing. However, there was retention of a band in the 38–39 kDa region, consistent with heat-stable tropomyosin.

**Fig 2:**
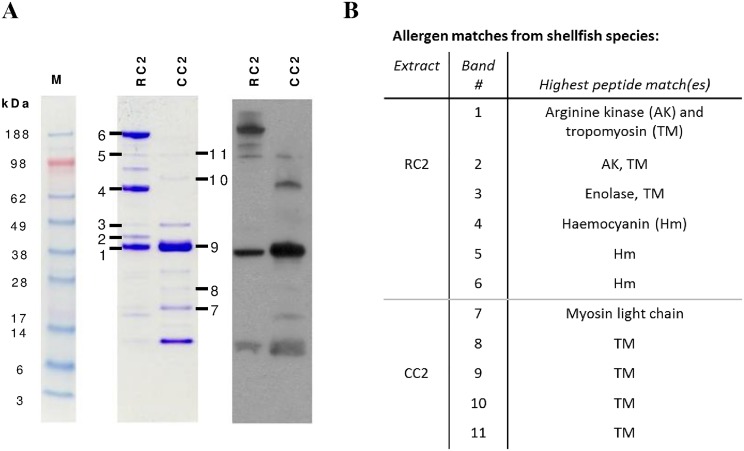
Mass spectrometric analysis of IgE-reactive proteins from mud crab extracts. (A) Coomassie stained SDS-PAGE and IgE immunoblot of proteins from raw mud crab (RC2) and cooked mud crab (CC2) extracts. M, molecular weight markers (kDa). (B) Mass spectrometric analysis for protein bands (numbered 1 to 11) summarised with highest peptide matches with crustacean shellfish species shown.

To assess whether allergen content contributed to differences in IgE reactivity, IgE reactive proteins within the raw and cooked mud crab extracts were compared using mass spectrometry (Fig 2). In the raw extract (RC2), multiple allergens were identified including arginine kinase, tropomyosin, enolase and haemocyanin. In contrast, many of the IgE-reactive bands in the cooked extract (CC2) were found to be tropomyosin, identified at 38–39 kDa, as aggregates (78 and 102 kDa) and as a fragment (25 kDa).

### Cooked extracts were more IgE reactive than raw extracts

**Fig 3:**
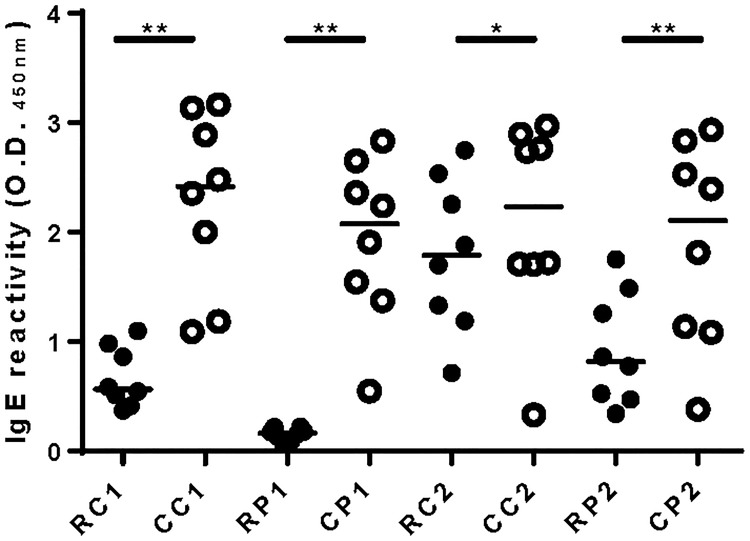
ELISA for serum IgE reactivity to crustacean extracts. Serum IgE reactivity to raw (closed symbols) and cooked (open symbols) crustacean extracts was tested by ELISA. IgE reactivity to raw blue swimmer crab (RC1), cooked blue swimmer crab (CC1), raw black tiger prawn (RP1), cooked black tiger prawn (CP1), raw mud crab (RC2), cooked mud crab (CC2), raw banana prawn (RP2), cooked banana prawn (CP2) extracts is shown for 8 crustacean-allergic subjects with median value indicated by bar. * p <0.05, ** p <0.01 (Wilcoxon matched-pairs signed rank test).

IgE ELISA showed that, for all species, cooked extracts had significantly higher IgE binding than the corresponding raw extracts (Fig 3). While black tiger prawn (RP1) bound significantly lower levels of IgE than other raw extracts (p <0.01), there were no significant differences in IgE binding between any of the cooked extracts. The mud crab extract IgE immunoblot result is consistent with the ELISA finding of greater IgE reactivity of cooked extract, in particular showing a marked increase in IgE reactivity of the 38–39 kDa tropomyosin band on cooking (Fig 2A).

### Crustacean-allergic and non-atopic subjects showed similar lymphocyte proliferative responses to crustacean extracts but with greater responses to raw extracts

**Fig 4:**
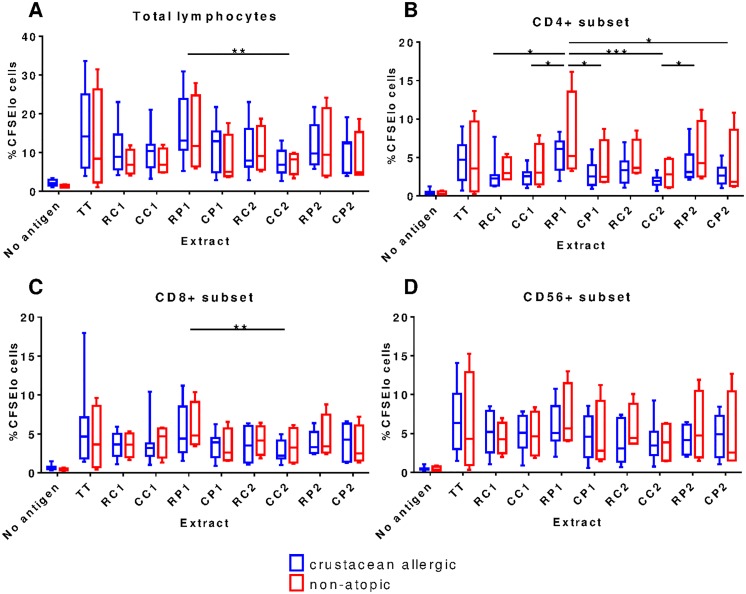
Proliferative response of PBMC lymphocytes to crustacean extracts. Roliferation of total (A), CD4^+^ (B), CD8^+^ (C) and CD56^+^ (D) lymphocytes in response to raw blue swimmer crab (RC1), cooked blue swimmer crab (CC1), raw black tiger prawn (RP1), cooked black tiger prawn (CP1), raw mud crab (RC2), cooked mud crab (CC2), raw banana prawn (RP2), cooked banana prawn (CP2) extracts is shown as assessed by CFSE staining at day 7 of culture. ‘No antigen’ was used as a negative control and tetanus toxoid (TT) as a positive indicator of proliferation. Box and whisker plots of crustacean-allergic (blue; n = 8) and non-atopic (red; n = 4) subject data represent minimum to maximum (whiskers), interquartile range (box) and median (bar) values (based on maximal proliferation for either 50 or 100 μg/mL extract). * p <0.05, ** p <0.01, *** p <0.001 (Friedman’s test with Dunn’s post hoc correction, combined allergic and non-atopic data).

To assess cellular immune responses to crustacean extracts, PBMC proliferative responses were first examined. Extract concentrations of 50 and 100 μg/mL, and 7 days of culture were found to be optimal for reliable detection of lymphocyte proliferation for raw and cooked extracts in preliminary experiments (data not shown). Crustacean extracts induced marked total lymphocyte proliferation above that observed in ‘no antigen’ control cultures (Fig 4A). Interestingly, crustacean-allergic and non-atopic subjects showed similar levels of PBMC proliferation in response to crustacean extracts with a trend for lower proliferation to cooked extracts than the corresponding raw extract, except for the blue swimmer crab.

**Fig 5:**
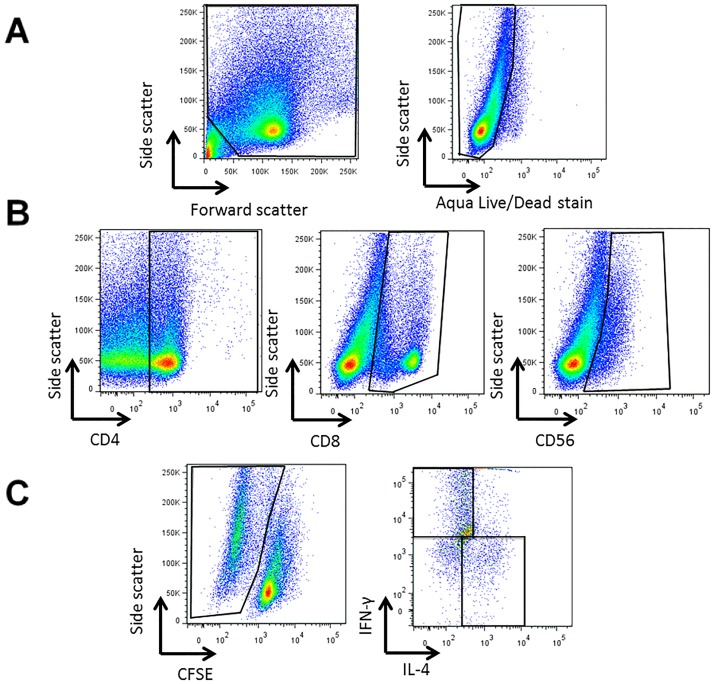
Representative flow cytometry gating strategy to assess immune cell response to crustacean extracts. A representative flow cytometry gating strategy for analysing proliferation and cytokine profiles of lymphocyte subsets in crustacean extract-stimulated PBMC cultures is shown. Cells were first gated on live, total lymphocytes (A), then CD4^+^, CD8^+^ or CD56^+^ lymphocytes (B). Next, proliferated (CFSElo) cells for each subset (e.g. CD8^+^) were identified and analysed for intracellular IL-4 and IFN-γ expression (C).

Next, proliferation of CD4^+^, CD8^+^ and CD56^+^ lymphocyte subsets was selectively analyzed (gating strategy in Fig 5). As for total lymphocytes, CD4^+^ cell proliferation was similar for crustacean-allergic and non-atopic subjects with a trend for lower proliferation to cooked extracts for all species except CC1 (Fig 4B). When data from all subjects were combined, the RP1 extract induced significantly higher proliferation compared to all of the cooked extracts. This result is in contrast to IgE binding results where RP1 had the lowest IgE binding of any extract. The reasons for the differing T cell proliferative and IgE reactivity of the RP1 extract are unclear, as we have shown previously that the allergen composition of prawn and crab extracts were very similar, with allergens from these species highly conserved between different crustacean species [4]. Proliferation of CD8^+^ and CD56^+^ subsets was observed in response to the crustacean extracts, but again there was no difference between crustacean-allergic and non-atopic subjects (Fig 4C and 4D). There were also no significant differences in CD8^+^ and CD56^+^ lymphocyte proliferation induced by raw and cooked extracts when all subject data were combined, except for RP1 that induced significantly higher CD8^+^ lymphocyte proliferation than CC2 (Fig 4C) (p <0.01).

### Cytokine profiles in crustacean extract-stimulated cultures showed a higher CD4^+^ cell IL-4^+^/IFN-γ^+^ ratio for crustacean-allergic subjects, but were not affected by cooking

**Fig 6:**
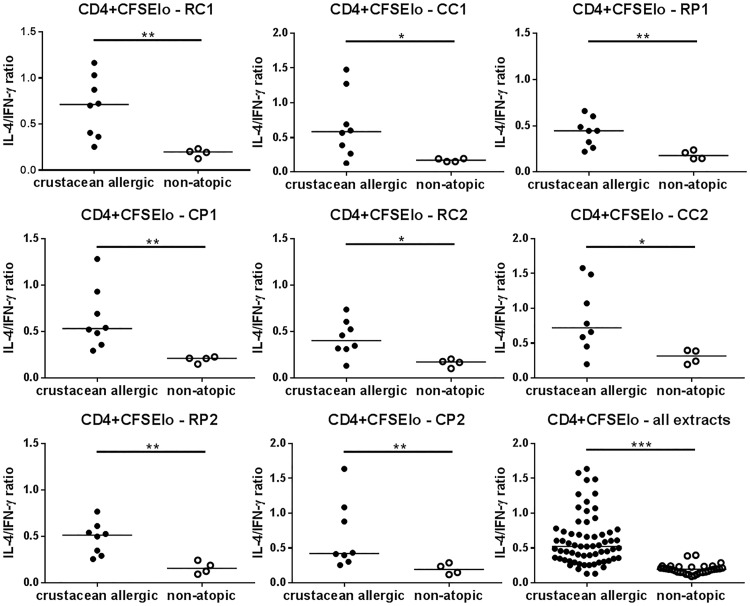
Cytokine profiles of PBMC stimulated with crustacean extracts. The ratio of percentage intracellular IL-4^+^/IFN-γ^+^ cells within proliferated (CFSElo) CD4^+^ lymphocytes in response to raw blue swimmer crab (RC1), cooked blue swimmer crab (CC1), raw black tiger prawn (RP1), cooked black tiger prawn (CP1), raw mud crab (RC2), cooked mud crab (CC2), raw banana prawn (RP2), and cooked banana prawn (CP2) extracts is shown for crustacean-allergic (n = 8; closed circles) and non-atopic subjects (n = 4; open circles). Maximal cytokine values for either 50 or 100 μg/mL extract used for ratio calculation. Bars represent median values. * p <0.05, ** p <0.01, *** p <0.001 (Mann Whitney test).

Analysis of the intracellular cytokine profile of lymphocytes proliferating in response to crustacean extracts showed an inverse relationship between the percentage IL-4^+^ and IFN-γ^+^ cells within each of the CD4^+^, CD8^+^ and CD56^+^ subsets. Particularly for the CD4^+^ subset, allergic subjects showed a higher percentage of IL-4^+^ cells and a lower percentage of IFN-γ^+^ cells compared with non-atopic subjects (Table 2; Fig 5 shows gating strategy). This Th2 polarization by CD4^+^ cells for allergic compared to non-atopic subjects is more clearly seen when data are presented as IL-4^+^/IFN-γ^+^ cell ratios to correct for variation between subjects in individual cytokine production [25](Fig 6). There was a similar trend for the CFSE^lo^ CD56^+^ subset, reaching statistical significance for CP1 (p <0.01) and RC2 (p <0.05) extracts (data not shown), but differences were less marked and not significant for the CFSE^lo^ CD8^+^ subset. Cooking did not significantly affect cytokine reactivity of the extracts for either subject group.

**Table 2:** Summary of cytokine production by proliferating (CFSE^lo^) lymphocytes in response to crustacean extracts for crustacean-allergic and control non-atopic subjects.

	Lymphocyte subset	CD4^+^		CD8^+^		CD56^+^	
	Atopic status	A	NA	A	NA	A	NA
**%IL-4**	**RC1**	29.5	13.6	14.2	11.5	27.5	16.5
		(14–41)	(10–16)	(7–32)	(6–14)	(19–41)	(13–27)
	**CC1**	22.5	11.5	12.9	11.4	25.5	19.6
		(8–38)	(10–15)	(6–27)	(11–14)	(13–41)	(12–24)
	**RP1**	23.7	12.2	15.9	12.3	26.5	19.9
		(14–34)	(11–15)	(9–20)	(9–22)	(12–32)	(17–31)
	**CP1**	23.5	14.4	18.8	13.3	33.7	19.3
		(17–43)	(10–17)	(10–29)	(10–20)	(21–48)	(18–23)
	**RC2**	22.4	12.4	14.2	10.3	30.7	20.2
		(9–34)	(8–14)	(5–32)	(8–20)	(21–42)	(11–28)
	**CC2**	28.4	18.1	18.3	18.7	31.4	34.5
		(11–45)	(12–24)	(10–49)	(15–27)	(22–53)	(21–45)
	**RP2**	26.2	12.0	16.4	10.3	26.1	18.9
		(16–37)	(7–16)	(10–46)	(9–21)	(22–62)	(12–32)
	**CP2**	27.0	12.5	12.2	16.7	30.9	20.1
		(15–43)	(9–20)	(6–35)	(11–32)	(12–54)	(9–41)
**%IFN-γ**	**RC1**	42.6	70.1	69.9	66.6	61.5	68.3
		(36–57)	(63–78)	(44–79)	(62–83)	(46–84)	(62–69)
	**CC1**	43.7	64.5	66.4	58.8	60.6	62.0
		(26–61)	(62–73)	(37–74)	(55–72)	(47–72)	(59–66)
	**RP1**	55.0	67.6	70.5	71.3	60.8	64.6
		(46–64)	(61–74)	(51–80)	(63–72)	(54–79)	(63–65)
	**CP1**	43.2	70.5	66.2	69.4	56.6	69.8
		(34–59)	(63–73)	(29–73)	(61–76)	(44–67)	(65–73)
	**RC2**	52.9	74.8	69.1	76.0	59.9	68.9
		(43–65)	(63–81)	(38–80)	(63–78)	(44–68)	(63–71)
	**CC2**	35.2	62.2	64.4	56.6	53.7	59.9
		(23–56)	(50–66)	(26–71)	(50–74)	(33–63)	(53–65)
	**RP2**	52.2	74.7	70.5	72.2	62.3	67.0
		(43–62)	(63–79)	(54–80)	(70–74)	(51–68)	(63–71)
	**CP2**	54.5	68.5	69.7	68.3	60.2	69.2
		(26–69)	(61–74)	(35–78)	(52–71)	(31–69)	(59–75)

Median values shown with ranges in parentheses; A = crustacean-allergic subjects (n = 8); NA = non-atopic subjects (n = 4). Boxes shaded grey indicate statistically significant differences (p<0.05 in light grey, p<0.01 in dark grey; Mann-Whitney test) between crustacean-allergic and non-atopic subjects for a particular lymphocyte subset and extract (raw blue swimmer crab (RC1), cooked blue swimmer crab (CC1), raw black tiger prawn (RP1), cooked black tiger prawn (CP1), raw mud crab (RC2), cooked mud crab (CC2), raw banana prawn (RP2), cooked banana prawn (CP2)). No significant difference was observed between raw and cooked extracts.

### CD4^+^ intracellular IL-4^+^ proportions correlated with total and specific IgE levels

**Fig 7:**
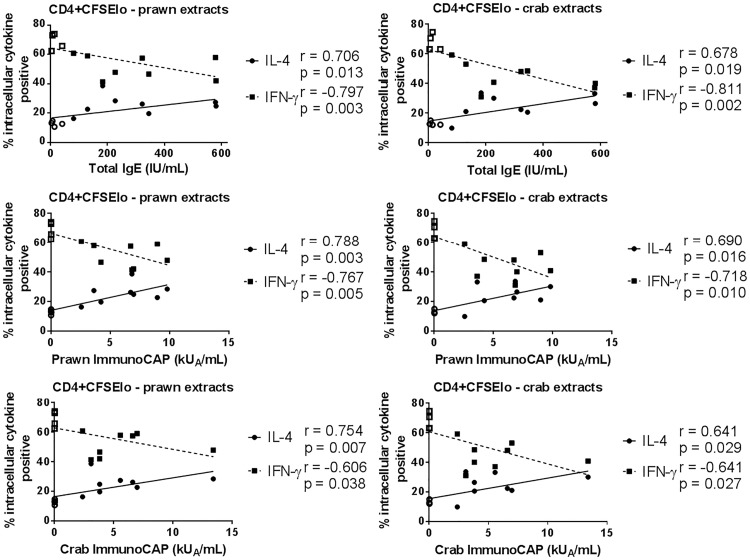
Correlation of total and specific IgE levels with cytokine response of CD4^+^ lymphocytes to crustacean extracts. Correlation of total, prawn-specific and crab-specific IgE levels (ImmunoCAP) with percentage intracellular IL-4^+^ (solid line) and IFN-γ^+^ (broken line) cells within proliferated (CFSElo) CD4^+^ lymphocytes in response to prawn or crab extracts for crustacean-allergic (closed symbols) and non-atopic (open symbols) subjects. For each subject, median percentage of cytokine positive cells in response to raw and cooked extracts from the two prawn species or two crab species are shown. Data analysed using a Spearman rank correlation test; rho (r) and p-values shown.

There was a significant positive correlation between total IgE and specific IgE levels and the percentage of IL-4^+^ cells within the proliferating CD4^+^ subset in response to prawn and crab extracts (for each subject, data were combined for all prawn extracts (RP1, CP1, RP2, CP2) and all crab extracts (RC1, CC1, RC2, CC2) to calculate the median percentage of cytokine positive cells; Fig 7). In the case of specific IgE levels, this correlation was stronger for prawn extracts than crab (p<0.01 vs p<0.05). Conversely, there was a significant negative correlation between total or specific IgE levels and the percentage of IFN-γ^+^ cells (p<0.05 or p<0.01). Similar relationships were observed for dividing CD56^+^ cells with regard to cytokine responses and total IgE, and IFN-γ responses and prawn-specific IgE (data not shown). There were no correlations between cytokines produced by dividing CD8^+^ cells and total or specific IgE. Proliferation of total and subsets of lymphocytes showed no correlation with total or specific IgE levels (data not shown).

### Cooking decreased the induction of regulatory T cells by crustacean extracts

**Fig 8:**
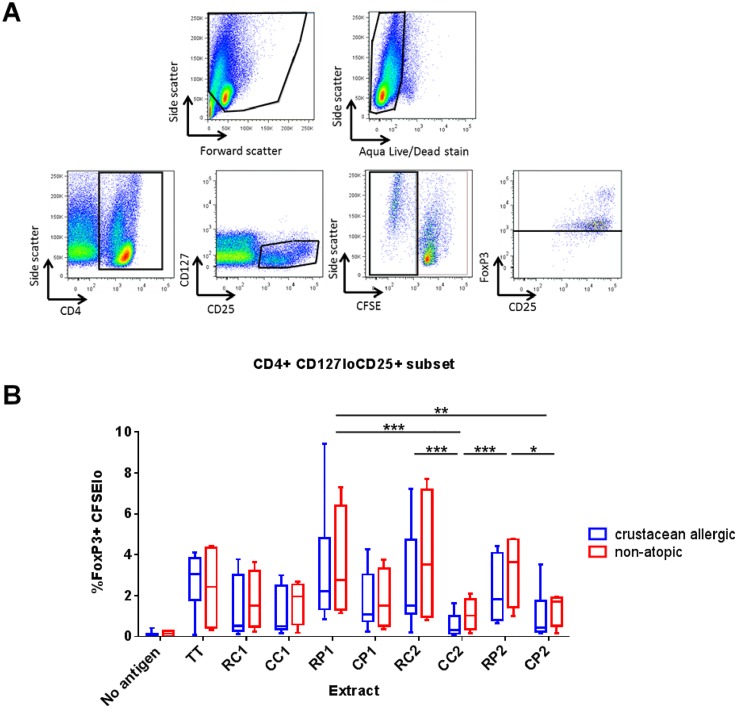
Regulatory T cell response to crustacean extracts. (A) Representative gating strategy for regulatory T cells (Tregs). Cells were first gated for live lymphocytes, then CD4^+^ lymphocytes. Next proliferated (CFSE^lo^) CD127^lo^CD25^+^ cells were analysed for FoxP3 expression. (B) Percentage of proliferated Tregs (CD4^+^CD127^lo^CD25^+^FoxP3^+^CFSE^lo^) in response to raw blue swimmer crab (RC1), cooked blue swimmer crab (CC1), raw black tiger prawn (RP1), cooked black tiger prawn (CP1), raw mud crab (RC2), cooked mud crab (CC2), raw banana prawn (RP2), cooked banana prawn (CP2) extracts at day 7 of culture. ‘No antigen’ was used as a negative control and tetanus toxoid (TT) as a positive control. Box and whisker plots of crustacean-allergic (blue; n = 8) and non-atopic (red; n = 4) subject data represent minimum to maximum (whiskers), interquartile range (box) and median (bar) values (based on maximal percentage of Tregs for either 50 or 100 μg/mL extract). Combined allergic and non-atopic subject data analysed using Friedman’s test with Dunn’s post hoc correction (* p <0.05, ** p <0.01, *** p <0.001).

Tregs were analysed based on a CD4^+^CD127^lo^CD25^+^Foxp3^+^ phenotype (gating strategy shown in Fig 8A). This phenotype (CD4^+^CD25^+^CD127^lo^FoxP3^+^) has been shown previously to correspond with functional Tregs [26, 27]. Within the non-dividing, CFSE^hi^ subset, cells with a Treg phenotype showed no differences in proportions in response to any of the extracts or between subject groups, and proportions were similar to those seen for the ‘no antigen’ control (data not shown). However, a wider range of Treg proportions was seen within the CFSE^lo^ cell population and some interesting differences observed. Non-atopic subjects displayed a trend for higher proportions of Tregs in response to each of the crustacean extracts, and Treg proportions were generally lower in cultures stimulated with cooked extracts compared with raw for crustacean-allergic and non-atopic subjects (Fig 8B).

## Discussion

Crustacean allergy is a serious health issue, but still poorly studied compared with other food allergies. Since crustaceans are frequently eaten after cooking, the effects of heating on crustacean allergenicity are important to determine in order to define reliable diagnostic markers and suitable preparations for specific immunotherapy. In agreement with our previous reports on the blue swimmer crab and black tiger prawn [4, 23], we showed a marked increase in IgE reactivity following cooking of all four crustacean species studied. However, there were contrasting results for immune cell reactivity. There was a trend for decreased PBMC proliferation in response to cooked crustacean extracts. Induction of Tregs was also decreased. However cooking did not substantially affect the effector phenotype of responding cells. For both raw and cooked extracts, there was a significant bias for CD4^+^ cells from crustacean-allergic subjects to produce the Th2 cytokine IL-4 rather than the Th1 cytokine IFN-γ, and the proportion of IL-4^+^ proliferating CD4^+^ cells correlated significantly with serum levels of total IgE and both prawn and crab specific-IgE antibody.

In this study, we used whole crustacean extracts to investigate the effects of thermal processing on humoral and cellular reactivity rather than recombinant or purified allergens since it has been shown that there are food-matrix related effects of heating [16, 17]. Whole extracts also present the mixture of allergens encountered naturally in food. We demonstrated that the heating protocol used in this study induced marked effects on the protein profiles and IgE reactivity of the crustacean extracts, similar to those shown previously by us and others [4, 21, 23, 28, 29]. There was loss of higher molecular weight proteins and an increase in lower molecular weight proteins, consistent with thermal degradation. Due to the paucity of information on allergens of the mud crab, we selected this species for follow-up mass spectrometric analysis of allergen content in raw and cooked extracts. This investigation showed that the high molecular weight band corresponding to haemocyanin in the raw extract was not observed in the cooked extract. Arginine kinase was also not identified within the cooked mud crab extract, consistent with being heat labile as documented previously for other crab species [30, 31]. However, there was also evidence for heat stable proteins such as tropomyosin, with mud crab tropomyosin retaining IgE reactivity as a monomer, aggregate and fragment in the cooked extract. The maintenance of IgE reactivity of tropomyosin when aggregated or partially degraded by cooking is in keeping with a previous study by us on the black tiger prawn which demonstrated the formation of IgE-reactive tropomyosin multimers and/or fragments in response to thermal processing [23].

Many of the bands in the heated preparations were smeared, possibly due to breakdown products but also glycation. The formation of advanced glycation end-products (AGE) following heating in the presence of endogenous or exogenous sugars by the Maillard reaction (non-enzymatic attachment of sugars to proteins) has been shown to increase IgE binding and T cell reactivity to other food allergens [18, 32]. Glycation may contribute to at least part of the modifications of crustacean proteins following heating and may promote the increase in IgE reactivity of the cooked crustacean extracts observed in this study. The mixed effects of heating on integrity and IgE reactivity of crustacean proteins observed here and our previously demonstrated wide range of IgE reactivity to different crustacean proteins in raw and heated extracts between subjects [4, 23], argue for inclusion of both raw and heated extracts and/or allergens in diagnostic assays for crustacean allergy. This will give greater accuracy in identifying crustacean-sensitized individuals, who are potentially at risk of severe allergic reactions such as anaphylaxis.

Despite the clear changes in protein profiles and IgE reactivity of crustacean extracts induced by heating, there were no marked differences in PBMC proliferative responses to raw versus cooked extracts. In fact there was a trend for greater cellular reactivity of the raw extracts. This could in part be explained by the difference in allergen composition between raw and cooked extracts, with raw extracts being shown in this study to contain a large number of individual allergens whilst cooked extracts predominantly contain tropomyosin. Therefore the raw extracts may be able to stimulate a wider repertoire of allergen-specific T cells than cooked extracts. To date only CD4^+^ T cell reactivity to shrimp tropomyosin has been studied [6]. Further study regarding the ability of individual crustacean allergens to stimulate T cells is required. However it should be noted that when testing of the effects of heating, the influence of the food matrix should be considered. Previous studies on purified tropomyosin show that high temperature alone does not alter IgE reactivity greatly unless it is in the presence of other proteins and/or sugars [17].

Our findings suggest that several prawn and crab allergens are able to induce good T cell responses, since PBMC proliferative responses to the mixed allergen content of raw extracts was similar to that for the ‘tropomyosin-enriched’ cooked extracts. The effect of cooking on allergen uptake by innate immune cells and the very different nature of T and B cell antigen epitopes also need to be considered, T cell epitopes comprising short linear peptides produced by APC processing while B cell epitopes are often conformational. The heat-induced changes that increased IgE reactivity of crustacean allergens apparently had no substantial effect on the net allergen peptide presentation to CD4^+^ or CD8^+^ T cells. We have previously demonstrated that patients generate IgE antibodies against both heat stable and heat labile allergens of crustaceans, with the heat stable tropomyosin being a major allergen [4, 21]. The relative importance of tropomyosin as a T cell-reactive allergen compared with other crustacean allergens remains to be demonstrated.

As noted previously for other food allergens [33, 34], there was no significant difference in lymphocyte proliferation between crustacean-allergic and non-atopic subjects in response to any of the crustacean extracts. This is not unexpected because, regardless of atopic status, subjects may have allergen-specific T cells in their blood that can proliferate *in vitro* in response to allergen-derived peptides presented by APC [35–37]. Arguably, of more clinical importance is the resultant effector phenotype of allergen-stimulated T cells as this is what ultimately directs the type of immune response and generation of allergen-specific IgE antibody.

To assess effector cell phenotype, the intracellular cytokines IL-4 and IFN-γ were analysed in CD4^+^, CD8^+^ and CD56^+^ lymphocyte subsets from crustacean-allergic and non-atopic subjects. CFSE^lo^ proliferating cells were analysed as these were the cells, either directly or indirectly, responding to *in vitro* stimulation with crustacean proteins. As expected, the production of IL-4 by dividing CD4^+^ lymphocytes was higher for crustacean-allergic subjects than non-atopic subjects, and vice versa for production of IFN-γ. These differences were shown more clearly by comparing IL-4^+^/IFN-γ^+^ ratios, better reflecting the Th2 polarisation of the immune response to allergen by allergic individuals and Th1 bias by non-atopics, and supported by the strong correlation with clinically relevant serum prawn- or crab-specific IgE levels. Proliferating CD8^+^ and CD56^+^ lymphocytes were also shown to contribute to the cytokine milieu in raw and cooked crustacean-stimulated PBMC cultures. Further studies are required to determine the precise phenotype of the CD8^+^ and CD56^+^ lymphocyte subsets responding (including use of a wider range of NK and NKT cell markers) and whether their responses were directly in response to extract stimulation or bystander effects from cytokines produced by the allergen-responsive CD4^+^ T cells in culture. Of particular interest, in terms of influencing clinical outcome, would be determining whether the whole crustacean extracts contain NK or NKT cell ligands that could engage directly with these cell subsets and stimulate their production of Th2 polarising cytokines. Cytokine production by both NK and NKT cells have been shown to play a role in promoting development of the allergic immune response [38–41].

Better defined is the role of Tregs in mediating immune tolerance to allergens [15, 36]. Typically, non-atopic subjects exhibit a stronger suppressive Treg response to allergen than allergic subjects [42]. Tregs have also been shown to play a role in clinically effective allergen-specific immunotherapy (see review [13]). In this study, we report for the first time the induction of Tregs by crustacean allergens by analysing the proliferating (CFSE^lo^) population of CD4^+^CD25^+^CD127^lo^Foxp3^+^ cells in allergen-stimulated PBMC cultures. This phenotype has been shown to correspond with functional Tregs [13]. There was a trend for a higher percentage of proliferated Tregs in response to each crustacean species for non-atopic subjects compared with crustacean-allergic subjects. Of note in this study, cooking generally decreased induction of Tregs by crustacean extracts.

Together, our findings inform the development of more reliable diagnostic assays for crustacean allergy as well as the design of an appropriate allergen-specific therapeutic. Conventional allergen immunotherapy using whole allergen is likely not suitable for treating crustacean allergy due to the high risk of IgE-mediated adverse reactions [43]. The most promising specific treatment option for allergens of high anaphylactic potential comprises peptides based on dominant CD4^+^ T cell epitopes of allergens [43, 44]. Our findings suggest that allergens from raw crustacean extracts will be suitable for generating relevant T cell lines and clones for comprehensive T cell epitope mapping for a peptide therapeutic. T cell reactivity to other crustacean allergens as well as tropomyosin needs to be assessed. Effector cell assays developed in this study also provide the basis for bioassays to monitor efficacy of treatment.
